# Rigor prophylaxis in stage IV melanoma and renal cell carcinoma patients treated with high dose IL-2

**DOI:** 10.1186/s12885-018-4810-y

**Published:** 2018-10-20

**Authors:** Brian Khong, Benjamin O. Lawson, Junjie Ma, Cheryl McGovern, Joan K. Van Atta, Abhijit Ray, Hung T. Khong

**Affiliations:** 1 Adventist Health White Memorial, 1720 East Cesar E Chavez Avenue, Los Angeles, CA 90033 USA; 2 Honor Health Medical Center, 9003 E Shea Blvd, Scottsdale, AZ 85260 USA; 3 Department of Pharmacotherapy, University of Utah, 30 S 2000 E, Salt Lake City, UT 84112 USA; 4 Huntsman Cancer Institute, University of Utah, 2000 Cir of Hope Dr, Salt Lake City, UT 84103 USA; 5 Department of Breast Oncology, Moffitt Cancer Center, 10920 McKinley Dr, Tampa, FL 33612 USA

**Keywords:** High dose interleukin-2, Rigor prophylaxis, Melanoma, Renal cell carcinoma, Tramadol, Meperidine

## Abstract

**Background:**

Rigors are a significant adverse event during interleukin-2 (IL2) therapy for metastatic melanoma and renal cell carcinoma. Meperidine has been a mainstay for rigor prophylaxis but there is a paucity of data regarding possible alternatives.

**Methods:**

Ninety one patients receiving IL2 therapy for metastatic renal cell carcinoma and melanoma at Huntsman Cancer institute (HCI), Utah from May 2009 to October 2016 were retrospectively evaluated for rigor prophylaxis. Forty two patients received meperidine and 49 received tramadol. Rigors were tabulated using the proxy of number of doses of as needed (PRN) rigor medications and normalized by IL2 doses. Other outcomes of fever, hypotension, and renal insufficiency were noted on a binary scale and normalized by cycles. Statistical analysis was performed utilizing univariate and multivariate negative binomial models.

**Results:**

Ninety one patients were identified with metastatic melanoma or RCC who received high dose IL2 therapy. Forty two received meperidine and 49 received tramadol prophylaxis for rigors. Univariate negative binomial analysis shows incidence rate ratios (IRR): fever 0.41 (95% CI 0.28–0.62, *p*-value < 0.001), hypotension 1.7 (95% CI 1.11–2.61, *p*-value 0.015), renal insufficiency 0.58 (95% CI 0.35–0.98, *p*-value 0.041), rigors per all PRN meds 1.01 (95% CI 0.79–1.28, *p*-value 0.964), and rigors via opioid PRN meds 0.85 (95% CI 0.67–1.07, *p*-value 0.168). Multivariate negative binomial analysis shows IRR: fever 0.59 (95% CI 0.28–1.24, *p*-value 0.163), hypotension 0.93 (95% CI 0.43–2.03, p-value 0.864), renal insufficiency 1.1 (95% CI 0.52–2.32, p-value 0.807), rigors per al PRN meds 0.92 (95% CI 0.67–1.26, *p*-value 0.604), and rigors via opioid PRN 0.9 (95% CI 0.65–1.26, *p*-value 0.554).

**Conclusion:**

Univariate models indicated meperidine pre-treatment was associated with significantly lower rates of fever and renal insufficiency whereas tramadol was associated with significantly lower rate of hypotension. However, when controlled for demographics and other treatment differences, these differences were no longer significant.

## Background

From 2010 to 2014, 46,251 deaths occurred from melanoma of the skin and 68,119 deaths from renal cell cancer [1]. Per the 2017 NCCN guidelines, high dose interleukin-2 (HD IL2) therapy for metastatic melanoma is recommended as second line [2] and recommended as first line for predominant clear cell histology for Stage IV renal cell carcinoma [3]. HD IL2 administered as a single agent has proven to be one of the most effective regimens for metastatic renal cell carcinoma and melanoma to date [4]. However, while not a major dose limiting toxicity, rigors is an incredibly common adverse effect that negatively impacts patients’ quality of life.

Initially, administration of HD IL2 was associated with mortality rates up to 4% [5–7]. Although mortality rates have decreased substantially, IL2 therapy still causes significant dose-related morbidity [8]. Manifestations of IL2 toxicity occur in most organ systems, including the heart, lung, kidneys and central nervous system [4]. IL2 toxicity is mediated through lymphoid infiltration, a well-described capillary leak syndrome and the local effects of secondary cytokines [9]. The complex mechanism of action whereby IL2 induces capillary leak syndrome is postulated to involve a series of steps, including induction of circulating cytokines, such as tumor necrosis factor-alpha and other interleukins; generation of complement-activation products; neutrophil activation; and activation of endothelial-cell antigens [9]. This in turn results in capillary leak syndrome-associated hypovolemia causing renal insufficiency secondary to pre-renal azotemia [9]. The release of cytokines after IL2 administration has also been implicated as the cause of flu-like symptoms, such as fevers, chills, myalgias, and arthralgias [9].

Chills, fever, and malaise are among the most common and predictable adverse events associated with HD IL2 [4]. Typically, chills develop within 1 to 2 h of the first or second dose and are treated with repeated doses of meperidine and warm blankets [9]. Fever, which usually develops within 2 to 4 h of administration of the first or second dose and may reach 40.5 C and is usually treated with anti-pyretics such as acetaminophen as well as addition of non-steroidal anti-inflammatory drug (NSAID), indomethacin, which has also been advocated [9, 10]. Renal toxicity associated with high-dose IL2 is typical of prerenal azotemia and is commonly a result of hypotension, decreased intravascular volume, and/or impaired cardiac function [9]. Renal toxicity is most effectively managed by administering fluid boluses at the onset of oliguria, with a relative limit on the total volume of 1 to 1.5 L/d above maintenance needs [9].

Meperidine has been the mainstay for rigor prophylaxis [11]. Meperidine is predominantly a μ-receptor agonist and acts principally on the central nervous system [12]. Meperidine has been shown to be effective in eliminating post HD IL2 shivering or rigors and even considered the gold standard for the treatment of shivering [13]. It has seen extensive use for decades with the first mention in the 1980s for amphotericin chills [14].

However, rigors can persist and remain severe despite meperidine prophylaxis. Tramadol has been used as an alternative at the University of Utah. Tramadol acts similarly as a μ-opioid receptor agonist albeit more weakly with a longer half-life and has additional mechanistic effects such as partial inhibition of norepinephrine and serotonin uptake [12]. Prior studies in anesthesiology analyzing the treatment of post-operative shivers [15, 16], spinal anesthetic shivers [17], and post-epidural shivers [18] have shown at least comparable efficacy of tramadol to meperidine with some studies finding superiority [15] or fewer side effects [18]. Meta-analyses of general shiver treatment have shown comparable effects between the two medications in critical care setting [12].

## Methods

This was a retrospective cohort, chart review study performed at the Division of Oncology at Huntsman Cancer Institute of the University of Utah. The protocol was approved by the University of Utah Institutional Review Board who also formally waived ethics approval and consent. The study analyzed patients with metastatic renal cell carcinoma or metastatic melanoma receiving HD IL2 treatment with rigor premedication from September 2008 to December 2016. Demographic data, medical history, and treatment records were reviewed including data on age, gender, race, previous treatment, disease states and diagnoses, dates of HD IL2 treatment, and treatment adverse effects. Initial review identified 148 patients received IL2, of which 137 received HD IL2 treatment. The study population was parsed further to 91, removing those who did not receive sufficient opioid prophylaxis or missed sufficient clinical documentation. For rigor premedication, 42 patients received meperidine and 49 received tramadol.

Patients undergoing HD IL2 therapy underwent a maximum of 4 courses, each constituting two 1-week-long cycles of IL2 dosing in the Intermediate Care Unit. For our study, we analyzed up to the first 4 cycles with actual number of doses and cycles completed dependent on toxicities and lack of disease progression. Patients received multiple premedications with each IL2 dose which included rigor prophylaxis in the form of meperidine or tramadol with variable adjuncts such as diphenhydramine, lorazepam, or morphine. As needed (PRN) medications used to treat rigor episodes included meperidine, morphine, hydromorphone, diphenhydramine, and lorazepam.

Pertinent adverse effect outcomes were rigors, fever, hypotension, and renal insufficiency. Rigor severity and frequency were recorded by proxy; tabulating total amount of as needed medication to resolve rigor episodes per cycle. Each dose of the as needed medications was given a score of one, summed, and then normalized by the number of IL2 doses. High fever was noted if there were two or more episodes of fever > = 101 F occurring at least twice within the hour of onset. Severe hypotension was noted if SBP < 90 that required pressors. Significant renal injury was noted if serum creatinine was 1.8 mg/dL or higher or oliguric at less than 10 ml/hr. within a 12 h period. Except for rigors, these were graded on a binary scale then normalized by number of cycles.

Statistical significance for all outcomes was determined via univariate regression analysis and then repeated with multivariate regression analysis to control for demographics and other possible confounding factors. Confounds controlled in multivariate regression analysis included age at IL2 inception, IL2 doses, race, gender, previous treatment, malignancy type, and additional tramadol therapy given at physician’s discretion. Probability < 0.05 was considered significant.

## Results

### Enrollment and base-line characteristics of the patients

Between May 2009 and October 2016, 91 patients who were pre-treated with either meperidine or tramadol prior to HD IL2 were identified. The patients in the two groups in the primary analysis had similar baseline characteristics (Table 1 and Table 2).

**Table 1:** Baseline characteristics with averages

	Meperidine^a^ (*N* = 42)		Tramadol^b^ (*N* = 49)		
	Mean	Sd	Mean	Sd	*p*-value
Age at IL2	51.52	9.25	53.90	9.04	0.220
IL2 dose	18.14	8.24	18.02	7.34	0.940

^a^Group 1 includes patients who were pretreated with meperidine

^b^Group 2 included those who were pretreated with tramadol

**Table 2:** Baseline characteristics with number of patients

	Meperidine^a^ (*N* = 42)		Tramadol^b^ (*N* = 49)		
	*N* pts	%	*N* pts	%	*p*-value
Race					0.177
White	43	88%	36	86%	
Hispanic	3	6%	4	10%	
American Indian	3	6%	0	0%	
Unknown	0	0%	2	5%	
Gender					0.232
Female	14	29%	17	40%	
Male	35	71%	25	60%	
Previous treatment	17	35%	7	17%	0.052
Malignancy					0.088
Renal Cell Carcinoma	39	80%	26	63%	
Melanoma	10	20%	15	37%	
Tramadol	10	24%	49	100%	< 0.001

^a^Group 1 includes patients who were pretreated with meperidine

^b^Group 2 included those who were pretreated with tramadol

Meperidine was given to 42 of the 91 patients with an average IL2 dose of 18.14 which was similar to those, average IL2 dose of 18.02, who received tramadol (*p*-value 0.940).

Tramadol was given to 49 of the 91 patients and of those 49 patients, 63% were diagnosed with Renal Cell Carcinoma and 37% were diagnosed with Melanoma. Whereas, 80% of the 42 patients that received meperidine were diagnosed with renal cell carcinoma and 20% were diagnosed with melanoma.

More patients in the meperidine group received previous treatment compared to patients in group 2 (35% vs 17%, *p*-value 0.052).

Furthermore, 24% patients that were pretreated with meperidine, actually received tramadol at some point during their treatment of high dose IL2 therapy (Table 2).

### Outcomes

Without controlling for demographics, patients pre-treated with meperidine demonstrated less incidence of post-IL2 fever and renal insufficiency (IRR 0.41, 95% CI 0.28–0.62, *p*-value < 0.001 and IRR 0.58, 95% CI 0.35–0.98, *p*-value = 0.041). On the contrary, pre-treatment with tramadol was found to be superior to preventing hypotension (IRR 1.70, 95% CI 1.11–2.61, *p*-value = 0.015). Rigor prophylaxis was equivocal (Table 3).

**Table 3:** Results of univariate negative binomial models

	IRR^a^	95% CI Upper	95% CI Lower	*p*-value
Fever	0.41	0.28	0.62	< 0.001
Hypotension	1.70	1.11	2.61	0.015
Renal insufficiency	0.58	0.35	0.98	0.041
Rigors via all PRN meds^b^	1.01	0.79	1.28	0.964
Rigors via opioid PRN med^c^	0.85	0.67	1.07	0.168

^a^Incidence Rate ratio

^b^Rigor severity/frequency tabulated via proxy PRN medication of opioids, benzodiazepines, and antihistamines

^c^Rigors severity/frequency tabulated via proxy PRN opioid medications only

Multivariate analysis showed different associations, and with control for confounds, the differences between the two groups were no longer significant. Patients who underwent IL2 therapy and pre-treated with meperidine had a lower rate of fever compared to those who were pre-treated with tramadol (IRR 0.59, 95% CI 0.28–1.24, *p*-value = 0.163). Pre-treatment with tramadol was equivocal compared to meperidine in preventing renal insufficiency when adjusting for the aforementioned variables (IRR 1.10, 95% CI, 0.52–2.32, *p*-value =0.807). When analyzing the prevention of rigors with all PRN meds versus only PRN opioids, meperidine was equivocal compared to tramadol (IRR 0.92, 95% CI 0.67–1.26, *p*-value = 0.604 and IRR 0.90, 95% CI 0.65–1.26, *p*-value = 0.554, respectively) (Table 4).

**Table 4:** Results of multivariate negative binomial models^a^

	IRR	95% CI Upper	95% CI Lower	*p*-value
Fever	0.59	0.28	1.24	0.163
Hypotension	0.93	0.43	2.03	0.864
Renal insufficiency	1.10	0.52	2.32	0.807
Rigors via all PRN meds	0.92	0.67	1.26	0.604
Rigors via opioid PRN med	0.90	0.65	1.26	0.554

^a^Adjusted for age at IL2, IL2 dose, race, gender, previous treatment, malignancy, and additional tramadol

^b^Incidence Rate ratio

^c^Rigor severity/frequency tabulated via proxy PRN medication of opioids, benzodiazepines, and antihistamines

^d^Rigors severity/frequency tabulated via proxy PRN opioid medications only

## Discussion

This is the first retrospective analysis examining alternatives to meperidine in HD IL2 patients for rigor prophylaxis as well as control of other adverse effects. IL2 therapy is associated with multiple adverse effects including rigor, fever, capillary leak, hypotension, and renal insufficiency due to its cytokine cascade [9]. While meperidine became the mainstay decades ago [9], there is a paucity of data regarding alternatives especially given that meperidine is renally excreted with the context of possible renal insufficiency associated with IL2 therapy. At the University of Utah, some providers had begun to supplant meperidine with tramadol thus prompting our investigation to possible differences. Tramadol, while mechanistically similar especially in metabolism and excretion, had been utilized with anecdotal superiority at Huntsman Cancer Institute possibly attributed to its longer half-life.

We examined 91 patients subdivided into two groups based on the primary rigor prophylaxis received. The scope was limited to meperidine and tramadol. Given that notation of rigor severity and frequency varied highly from patient to patient especially between different providers, as needed medications used afterwards to control rigors were tabulated as a suitable proxy for rigor severity/frequency. Because rigor treatment was highly variable, treatments ranged from opioids only to those who included diphenhydramine and lorazepam. Due to this heterogeneity, two data sets were parsed and analyzed separately with one set representing rigor proxy as opioid use only and another set with rigor treatment including opioids, benzodiazepines, and antihistamines. Other significant adverse effects noted above were observed for potential differences as well between the two groups.

Univariate analysis showed significantly lower rates of fever, and renal insufficiency in the meperidine group albeit with higher hypotension rates. Adjusting for age, dosage, race, gender, previous treatment, disease, and additional tramadol use obliterated these significant differences, showing only equivocality. These results imply tramadol would be a potentially reasonable alternative to meperidine for rigor prophylaxis.

### Limitations

The study had several limitations, especially in its scope, heterogeneity of care, and the quality of the data.

As multiple physicians and nurse practitioners supervised the care of the IL2 patients, treatment regimens and accuracy of recordkeeping varied highly. Rigors treatment between teams varied in terms of pre-medication and as needed medications. Differences included initial premedication choice, timing and frequency of premedication transitions, dosage sizes, and choice of as needed medication for particular rigor episodes. Treatment teams had distinct preferences, for example starting patients with meperidine pre-medication then switching to tramadol at later doses within cycles once rigors proved severe.

Records disparities were evident as well especially between clinical notes and medication records. Rigors noted in notes often did not match amounts of PRN medications actually ordered and administered. In some cases, notes mentioned multiple or severe rigors requiring multiple medications to resolve yet medication records showed few to no PRN medication for rigor resolution. This held true especially for records prior to late 2011. Given the disparity, the medication record was assumed to be more accurate.

Scoring for rigors via as needed medications was tabulated with equal scoring ascribed to each type regardless of narcotic type, dose size, and other PRN-drugs such as benzodiazepines and antihistamines. Dosage sizes of both pre-medications and as needed medications were not tabulated; only frequency was recorded irrespective of size. Likely such details of dose normalization and differentiation of rigor treatment medications would contribute to improved analysis of rigor control and affect our results. Additionally, given time restrictions, data on the other adverse effects of fever, hypotension, and renal insufficiency was tabulated in a simplified manner as noted above. This binary tabulation limits the detail by which we analyzed their severity and frequency.

## Conclusions

Tramadol appears to be a reasonable alternative to meperidine for rigor prophylaxis. Given the nonsignificant differences in adverse events of meperidine coupled with its long track record in this area, however, it may be beneficial to continue meperidine use. Further research is warranted to clearly observe the differences between the two especially in light of the limitations highlighted above. While severe rigors are rarely a dose limiting toxicity in our practice, proper management improves patient quality of life and remains an integral part of patient care.

## Abbreviations

CI: confidence interval
HCI: Huntsman Cancer Institute
HD IL2: high dose interleukin-2
IL-2: Interleukin-2
IRR: incidence rate ratio
NCCN: National Comprehensive Cancer Network
NSAID: non-steroidal anti-inflammatory drug
PRN: as needed
RCC: renal cell carcinoma
SBP: systolic blood pressure
